# Localising nuclear spins by pseudocontact shifts from a single tagging site

**DOI:** 10.5194/mr-3-65-2022

**Published:** 2022-05-09

**Authors:** Henry W. Orton, Elwy H. Abdelkader, Lydia Topping, Stephen J. Butler, Gottfried Otting

**Affiliations:** 1 ARC Centre of Excellence for Innovations in Peptide & Protein Science, Research School of Chemistry, Australian National University, Canberra, ACT 2601, Australia; 2 Department of Chemistry, Loughborough University, Epinal Way, Loughborough, LE11 3TU, United Kingdom

## Abstract

Ligating a protein at a specific site with a tag molecule containing a paramagnetic metal ion provides a versatile way of generating pseudocontact shifts (PCSs) in nuclear magnetic resonance (NMR) spectra. PCSs can be observed for nuclear spins far from the tagging site, and PCSs generated from multiple tagging sites have been shown to enable highly accurate structure determinations at specific sites of interest, even when using flexible tags, provided the fitted effective magnetic susceptibility anisotropy ( Δχ ) tensors accurately back-calculate the experimental PCSs measured in the immediate vicinity of the site of interest. The present work investigates the situation where only the local structure of a protein region or bound ligand is to be determined rather than the structure of the entire molecular system. In this case, the need for gathering structural information from tags deployed at multiple sites may be queried. Our study presents a computational simulation of the structural information available from samples produced with single tags attached at up to six different sites, up to six different tags attached to a single site, and in-between scenarios. The results indicate that the number of tags is more important than the number of tagging sites. This has important practical implications, as it is much easier to identify a single site that is suitable for tagging than multiple ones. In an initial experimental demonstration with the ubiquitin mutant S57C, PCSs generated with four different tags at a single site are shown to accurately pinpoint the location of amide protons in different segments of the protein.

## 1 Introduction

Pseudocontact shifts (PCSs), which are generated by paramagnetic metal ions with fast-relaxing electron spins, provide outstanding restraints for the structure refinement of biological macromolecules [Bibr bib1.bibx26]. To make full use of PCS restraints, a large number of metal tags have been developed in recent years with the express purpose to install a paramagnetic centre on proteins, measure PCSs, and gain structural information [Bibr bib1.bibx25]. With restraints from multiple tagging sites, PCSs of backbone amide protons have been shown to be sufficient for 3D structure determinations of proteins [Bibr bib1.bibx46]. Particularly appealing applications of PCSs have been the structure refinement of polypeptide segments in proteins of known 3D fold [Bibr bib1.bibx2] and the structural characterisation of the interfaces of protein–protein [Bibr bib1.bibx42] and protein–ligand complexes [Bibr bib1.bibx43], as the long-range nature of PCSs allows the attachment of the metal tags at a distance that is sufficiently far from the site of interest to avoid structural perturbations by the tag.

The present work investigates the optimal tagging strategy for determining structural detail at a selected local site in a protein (site of interest – SoI), assuming that the 3D structure of the rest of the protein is known. In this scenario, the PCSs observed for the structurally known part can be used to determine the parameters of the magnetic susceptibility anisotropy ( Δχ ) tensor associated with the paramagnetic tag, and the remaining PCSs can be used to determine the 3D structure of the SoI. Fundamentally, the same scenario applies when the binding mode of a ligand or the structure of a protein–protein interface is to be determined.

The structure determination problem can be approached in two principally different ways. (i) Different sites can be selected in the protein for tagging. Most metal tags are designed for ligation to the thiol groups of one or two cysteine residues, which can be introduced into the target protein by mutagenesis of each of the target sites. This is the most common strategy [Bibr bib1.bibx20]. (ii) A single site is selected in the protein and several different samples are prepared with different paramagnetic metal ions. This approach was applied to the N-terminal domain of the *E. coli* DNA polymerase subunit ϵ to elucidate the binding mode of a small ligand [Bibr bib1.bibx15] and the subunit θ [Bibr bib1.bibx42]. The approach proved successful, even though the precision of the solutions was affected by a fairly close alignment of the ( Δχ ) tensors. This strategy, however, can be generalised by the use of different tags capable of delivering significantly different orientations of the magnetic susceptibility tensors relative to the target protein. To the best of our knowledge, the relative performance of these tagging strategies in terms of defining local structure has not been explored systematically.

The simulations performed in the present work predict that the chances of obtaining good structural restraints at the SoI are almost equally good when the same tag is deployed at multiple sites compared to when a single site is furnished with different tags. To gain an understanding of this effect, we consider the way in which PCSs determine the positions of nuclear spins, develop metrics for determining the precision with which the nuclear spins at the SoI can be localised, and calculate the chances of obtaining good localisation precision by assuming different numbers of tags and tagging sites and that the relative orientations of the Δχ tensors cannot be predicted and arise by chance.

In a practical demonstration, we use PCSs generated by four different tags attached at residue 57 of ubiquitin S57C to determine the positions of amide protons in different polypeptide segments of the protein.

### 1.1 Localisation spaces

The position of a nuclear spin can be defined as the 3D space in which it is to be found according to the measured PCS values and their associated uncertainties. We refer to this space as the localisation space. The localisation space is defined by multiple PCSs associated with different Δχ tensors.

The PCS of a nuclear spin is manifested in nuclear magnetic resonance (NMR) spectra as the change in chemical shift observed in the paramagnetic state compared to a diamagnetic reference, which is the same compound produced with a diamagnetic metal ion. Equation ([Disp-formula Ch1.E1]) describes the PCS of the nuclear spin, where r is the electron–nuclear distance, x , y , and z are the nuclear coordinates relative to the paramagnetic centre which defines the origin of the coordinate system, and $\Delta \chi_{ij}$ denote the components of the Δχ tensor in matrix representation.

1 $$\begin{array}{rl} \delta & =\frac{1}{4\pi r^{5}}\left[\begin{array}{ccccc} x^{2}-z^{2} & y^{2}-z^{2} & 2xy & 2xz & 2yz \end{array}\right]\\ & \cdot \left[\begin{array}{c} \Delta \chi_{xx}\\ \Delta \chi_{yy}\\ \Delta \chi_{xy}\\ \Delta \chi_{xz}\\ \Delta \chi_{yz} \end{array}\right]. \end{array}$$

The Δχ tensor is characterised by eight parameters which define its position, anisotropy, and orientation. These parameters can be determined by fitting to experimentally measured PCSs of nuclear spins located in the structurally known part of the protein. Most often, the PCSs of backbone amide protons are used to determine the parameters of the Δχ tensor.

For each PCS value, the Δχ tensor defines a non-spherical isosurface, which describes the coordinates where this particular PCS value is observed. The PCS isosurfaces are point symmetrical about the metal ion. The PCS measured of a nuclear spin at the SoI thus pins its location to the associated PCS isosurface. A second PCS measured with a second tag introduces a second PCS isosurface as a restraint. Disregarding rare borderline cases, the intersection between two isosurfaces describes a line, the intersection with a third isosurface defines two points, and the intersection with a fourth PCS isosurface restricts the position of the nuclear spin to a single point. Taking into account the experimental uncertainties, the isosurfaces expand to shells, and the common point expands to a 3D localisation space. In this way, multiple known Δχ tensors allow the positions of individual atoms in a molecule to be determined.

### 1.2 Δχ tensor orthogonality

The determination of localisation spaces requires that PCS isosurfaces intersect, which is readily achieved by positioning metal tags at different sites of the protein. It is not always straightforward, however, to identify multiple tagging sites that are at an optimal distance from the SoI and suitable for chemical modification without significant impact on the structure and function of the target protein. The alternative strategy of varying the isosurfaces by exchanging the paramagnetic ion in the same metal binding site also produces largely unsatisfactory results. For example, it has commonly been observed that the coordinate systems of Δχ tensors associated with two different paramagnetic lanthanoid ions such as Tb(III) and Tm(III) are closely aligned if the tagging site and chemical structure of the tag are the same [Bibr bib1.bibx3]. Even if the respective Δχ tensors differ slightly in their relative orientation, their PCS isosurfaces are likely to intersect at a shallow angle. In this situation, the intersection line between the two tensors can shift a lot in response to small inaccuracies in the Δχ tensors, limiting the precision with which the localisation space can be determined. The situation is different, however, if different chemical tags are used to install the paramagnetic centres, as the coordinate systems of the associated Δχ tensors are more likely to be oriented differently than to align. Furthermore, the exact positions of the different tags tend to differ to some extent, even when they are attached at the same site.

This work aims to quantify the precision with which localisation spaces can be determined by PCSs, assuming a variable number of tagging sites on the protein, which are arranged in different geometries. To address the difficulty in predicting the relative orientations of the respective Δχ tensors, the analysis employed computational Monte Carlo methods to arrive at statistical likelihoods of successful determinations of precise localisation spaces.

## 2 Results

The boundaries of an experimentally determined localisation space are determined by uncertainties in the Δχ tensor fits and the error associated with each individual PCS measurement. In the following, the problem is simplified by assuming that the protein coordinates underpinning the Δχ fits are correct and the Δχ tensors free of uncertainties.

We consider two different metrics for measuring the precision with which the localisation space of a nuclear spin can be determined from PCSs arising from multiple Δχ tensors. The first metric involves a discrete integration on a grid to capture a volume which fulfils a root mean square deviation (RMSD) criterion of experimental versus calculated PCS values. The second metric considers the relative geometry of PCS gradient vectors at the site of interest.

### 2.1 RMSD volume metric

Given n different Δχ tensors, Eq. ([Disp-formula Ch1.E2]) calculates the RMSD between the experimentally measured PCS, $\delta_{\text{exp},i}$ , and the calculated PCS, $\delta_{\text{cal},i}$ , for the i th Δχ tensor. $\delta_{\text{RMSD}}$ can be evaluated at any position of the SoI by calculating $\delta_{\text{cal},i}$ from Eq. ([Disp-formula Ch1.E1]).

2 $$\delta_{\text{RMSD}}=\sqrt{\frac{1}{n}\sum_{i}^{n}{\left(\delta_{\text{exp},i}-\delta_{\text{cal},i}\right)}^{2}}.$$

By evaluating Eq. ([Disp-formula Ch1.E2]) over a 3-dimensional grid of points and identifying the total number of grid points with an RMSD below a given threshold ( $\delta_{\text{RMSD}}^{\text{thresh.}}$ ), a volume is obtained that encapsulates all solutions below the RMSD threshold. This definition is encompassed by Eq. ([Disp-formula Ch1.E3]), where the PCS RMSD volume $V_{\text{RMSD}}$ is calculated over a grid of points with the coordinates x , y , and z , and ΔV is the volume occupied by a single grid point.

3 $$V_{\text{RMSD}}=\sum_{x,y,z}\left\{\begin{array}{ll} \Delta V, & \delta_{\text{RMSD}}(x,y,z)<\delta_{\text{RMSD}}^{\text{thresh.}}\\ 0, & \delta_{\text{RMSD}}(x,y,z)\geq \delta_{\text{RMSD}}^{\text{thresh.}} \end{array}\right.\;.$$

The threshold RMSD $\delta_{\text{RMSD}}^{\text{thresh.}}$ can be chosen to reflect the experimental uncertainty in the measured PCS value. The RMSD volume $V_{\text{RMSD}}$ then describes the localisation space of the nuclear spin, where a small value means its position can be determined precisely.

### 2.2 PCS gradient metric

Considering a single Δχ tensor, the space of solutions, where the experimental and theoretical PCSs are the same, is defined by an isosurface. In the immediate vicinity of a point on the isosurface, the local environment is approximated by a plane which can be described by a vector normal to the surface. The vector is defined by the PCS gradient field $\nabla \delta_{\text{PCS}}$ , which can be written as shown in Eq. ([Disp-formula Ch1.E5]). The gradient vector also describes the direction in which the position of the nuclear spin can be determined with the greatest precision as it corresponds to the direction of maximum change in the predicted PCS value.

4∇δPCS=∂∂x∂∂y∂∂zδPCS5=14πr52xΔχxx+2yΔχxy+2zΔχxz2xΔχxy+2yΔχyy+2zΔχyz2xΔχxz+2yΔχyz-2zΔχxx+Δχyy-(x2-z2)Δχxx+(y2-z2)Δχyy+2xyΔχxy+2xzΔχxz+2yzΔχyz4πr7⋅5x5y5z.

Considering two or three Δχ tensors and using the intersection between the associated PCS isosurfaces to localise a nuclear spin, the most precise localisation is achieved when the PCS isosurfaces intersect in an orthogonal manner. A quantitative measure of orthogonality can be defined in different ways. For example, orthogonality can be characterised by the dot product of vectors, which is akin to the angle score developed by [Bibr bib1.bibx53]. Equation ([Disp-formula Ch1.E6]) defines the parallel metric $\delta_{∥}$ as the sum of the absolute values of the pairwise dot products of the normalised PCS gradient vectors v . Equation ([Disp-formula Ch1.E7]) is equivalent, where $\theta_{ij}$ is the angle between the gradient vectors $v_{i}$ and $v_{j}$ . The parallel metric can assume a value between 0 and 1 and is small for nearly orthogonal PCS gradients. Note that a value of zero can only be obtained when the number of Δχ tensors considered is three or less.

6δ∥=1n-1∑i,ji≠jnvi⋅vjvivj7=1n-1∑i,ji≠jncos⁡θij.

As the PCS scales with $r^{-3}$ , the steepest PCS gradients occur close to the paramagnetic centre. This information is contained in the PCS gradient vector $\nabla \delta_{\text{PCS}}$ but is discarded in Eq. ([Disp-formula Ch1.E6]) due to the normalisation of the vectors. To account for proximity to the paramagnetic centre, the perpendicular metric $\delta_{⟂}$ is proposed as defined by Eq. ([Disp-formula Ch1.E8]), which preserves the gradient vector magnitudes through a pairwise cross-product (denoted by the ∧ symbol). Equation ([Disp-formula Ch1.E9]) is equivalent, where $\theta_{ij}$ is the angle between the gradient vectors $v_{i}$ and $v_{j}$ .

8δ⟂=1n-1∑i,ji≠jnvi∧vj9=1n-1∑i,ji≠jn|sin⁡θij|vivj.

The perpendicular metric awards a large value to Δχ tensor geometries that produce large and orthogonal PCS gradient vectors. The metric has a lower bound of 0 and no upper bound.

### 2.3 Model of tagging geometries

To compare the accuracy with which localisation spaces can be determined by PCSs generated by a different number of tags positioned at a different number of sites, we assumed a simple model of a globular macromolecule with metal tags attached to its surface and performed Monte Carlo simulations to sample Δχ tensors (referred to in the following as tags) with fixed positions and anisotropy but random orientations. The tagging sites (identical to the location of each paramagnetic centre) were located on the surface of a sphere of radius 25 Å, chosen to represent the macromolecule. Tagging sites were placed at even divisions of a circle on the sphere, each at the same distance from the nucleus of interest (Fig. [Fig Ch1.F1]). Calculations were performed for different numbers of tagging sites ranging from 1 to 6. In addition, the multiplicity of tags at a given site was varied such that the tags were distributed between the tagging sites as evenly as possible. For example, with five tags and three tagging sites, one tag was at site 1 and two tags each at sites 2 and 3.

**Figure 1 Ch1.F1:**
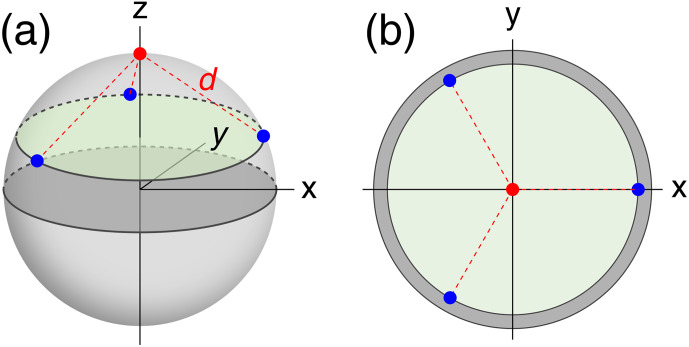
Example tagging geometry for three sites. The nuclear position of interest is located at the red dot, with the tagging sites located at the blue dots, equidistant about the circumference of the green disc. d denotes the distance of the nuclear spin from the paramagnetic centres. **(a)** View of the parameters used in a 3-dimensional representation. **(b)** View along the z axis onto the 2-dimensional cross-section containing the tagging sites.

### 2.4 Sampling tensor orientations

For any given geometry of sites and tag multiplicity, the tensor orientations were sampled from a random uniform distribution. The PCS RMSD volume was then calculated at the SoI using Eq. ([Disp-formula Ch1.E3]). This process was repeated 10 000 times to obtain a histogram depicting the frequencies with which different localisation volumes $V_{\text{RMSD}}$ were obtained. Figure [Fig Ch1.F2] shows smoothed representations of these distributions for a nuclear spin located 20 Å from each of the paramagnetic centres, calculated for $\delta_{\text{RMSD}}^{\text{thresh.}}$ of 0.03 ppm (parts per million), where the value of 0.03 ppm was chosen as representative of typical experimental uncertainties in PCS measurements of protein NMR signals.

**Figure 2 Ch1.F2:**
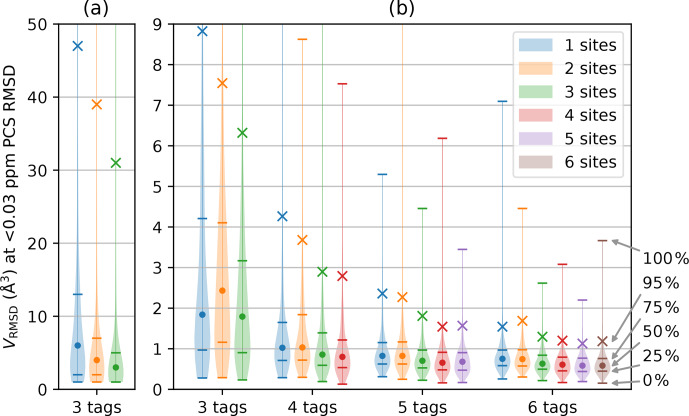
Distribution of $V_{\text{RMSD}}$ for Δχ tensors with 10 000 randomly sampled orientations and various tagging site geometries and multiplicities. The distance of the nuclear spin from the paramagnetic centre was 20 Å, and $\delta_{\text{RMSD}}^{\text{thresh.}}$ was set to 0.03 ppm. The widths of the lines in the horizontal dimension trace the histogram distributions of $V_{\text{RMSD}}$ . Percentiles indicated on the right identify the median of the distributions (labelled by a circle), the 95th percentile (marked by a cross) and the quartiles (identified by horizontal lines). **(a)** Calculations performed for a grid volume of $10^{6}$ Å ${}^{3}$ , with a point density of 1 point per Ångström, resulting in $10^{6}$ grid points and ΔV=1 Å ${}^{3}$ . **(b)** Calculations performed for a grid volume of 1000 Å ${}^{3}$ with a point density of 5 points per Ångström, resulting in 125 000 grid points and ΔV=0.008 Å ${}^{3}$ . The $V_{\text{RMSD}}$ values in panel **(a)** are increased due to the coarser point density used.

Figure [Fig Ch1.F2] summarises the results of the calculations for different numbers of tags and tagging sites. As expected, a greater number of tags improves the chances that the PCSs of the nuclear spin define a small localisation space. Furthermore, distributing a given number of tags over more tagging sites generally decreases the localisation volumes. Importantly, the calculations produced some non-intuitive results. (i) The advantage gained by a large number of tags is small. While the use of four instead of three tags may still deliver a significant reduction in the localisation space, the advantage gained by using more than four tags is likely to be very small. (ii) In general, using more tags is of greater benefit than using more tagging sites. This is evidenced by consistently smaller RMSD volumes associated with, e.g., the 95th percentile when using n+1 rather than n tags. (iii) Multiple different tags attached at the same site deliver localisation spaces only a little less confined than when the same number of tags is distributed over different sites. These findings have the potential to greatly simplify the structural characterisation of a specific SoI by PCSs, as it is much easier to install different tags in the same protein mutant than to identify a multitude of suitable tagging sites.

Closer inspection of Fig. [Fig Ch1.F2]b reveals a few irregularities arising from the specific geometries chosen and limitations associated with a finite grid. For example, the two-site geometry, which corresponds to a linear arrangement of tag–SoI–tag, was occasionally predicted to perform less well than the same number of tags located at a single site. The irregularity is particularly pronounced for the three distributions associated with the three-tag scenario in Fig. [Fig Ch1.F2]b. In this case, the largest localisation spaces (approximately the top 30 %) extended beyond the boundary of the grid so that their true volume sum $V_{\text{RMSD}}$ was larger than calculated and plotted. Although very large volumes can arise when PCS isosurfaces intersect at a shallow angle, the resulting localisation spaces feature long extensions, most of which may well be incompatible with the chemical structure of a SoI. Therefore, such extended localisation spaces may still present very useful structure restraints. More concerning is that the three-tag scenario also indicated that the median of the localisation space distribution was larger for two tagging sites than one. Closer inspection revealed that this anomaly was again caused by the limited grid volume used, which failed to capture every point below the RMSD threshold. The expected correlation between $V_{\text{RMSD}}$ and the number of tagging sites was re-established when the calculations were repeated with a larger grid volume, as shown in Fig. [Fig Ch1.F2]a.

While a finite grid size can adequately probe the environment of a SoI, anomalies were encountered when the PCS isosurfaces defined more than a single common intersection point. In 3D space, the PCS isosurfaces of four different Δχ tensors intersect at a single point, whereas the restraints posed by three PCSs can generally be fulfilled by two separate localisation spaces. Figure [Fig Ch1.F3] illustrates this well-known phenomenon [Bibr bib1.bibx21] by an example in 2D space, showing how two Δχ tensors produce two separate intersection points. Importantly, the intersection points are much better separated from each other if the tags are placed at the same location rather than at two different sites. As the calculations of Fig. [Fig Ch1.F2] used a finite grid size (symbolised by a grey-shaded box in Fig. [Fig Ch1.F3]), the three-tag calculations were more likely to capture both solutions in the two-site versus one-site simulations. Although this effect artificially disadvantages the two-site results in the calculations, it is nonetheless of practical relevancy, as a large separation between two possible localisation spaces more readily distinguishes one of the solutions as incompatible with the covalent structure of the molecule.

**Figure 3 Ch1.F3:**
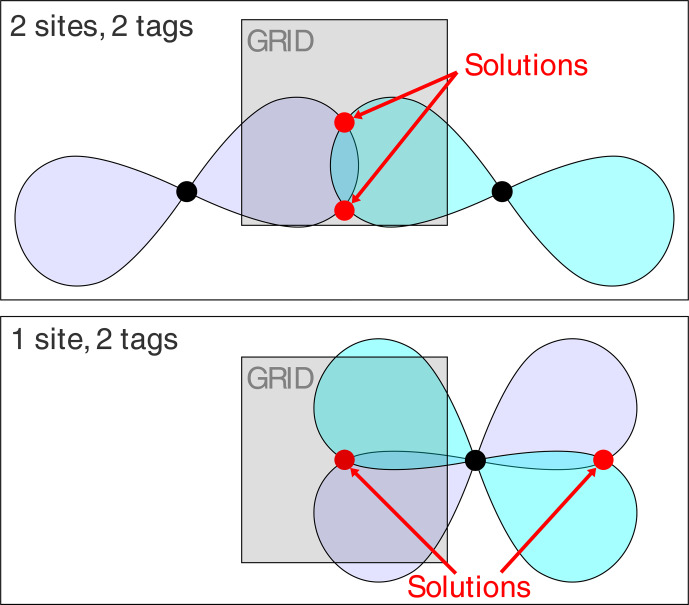
A 2-dimensional representation of isosurface intersection points for different tagging site geometries.

### 2.5 Assessing the quality of localisation spaces

The metrics of Eqs. ([Disp-formula Ch1.E8]) and ([Disp-formula Ch1.E6]) are designed to characterise the quality of a localisation space determination by a single number. Figure [Fig Ch1.F4] shows that the perpendicular metric tends to correlate with the localisation volume more closely than the parallel metric. In particular, the largest values of $\delta_{⟂}$ correlate very well with the smallest localisation volumes. Therefore, in the following, we mainly consider the perpendicular metric, but also note its limited information content, as the correlation between large $\delta_{⟂}$ values and small localisation volume breaks for small values of the metric. Notably, localisation spaces can assume very irregular shapes, which include points entirely incompatible with the covalent structure of the molecule. In many cases, the attempt to characterise the quality of a localisation space by a single one of the metrics above very much underestimates the quality of the actual localisation space.

**Figure 4 Ch1.F4:**
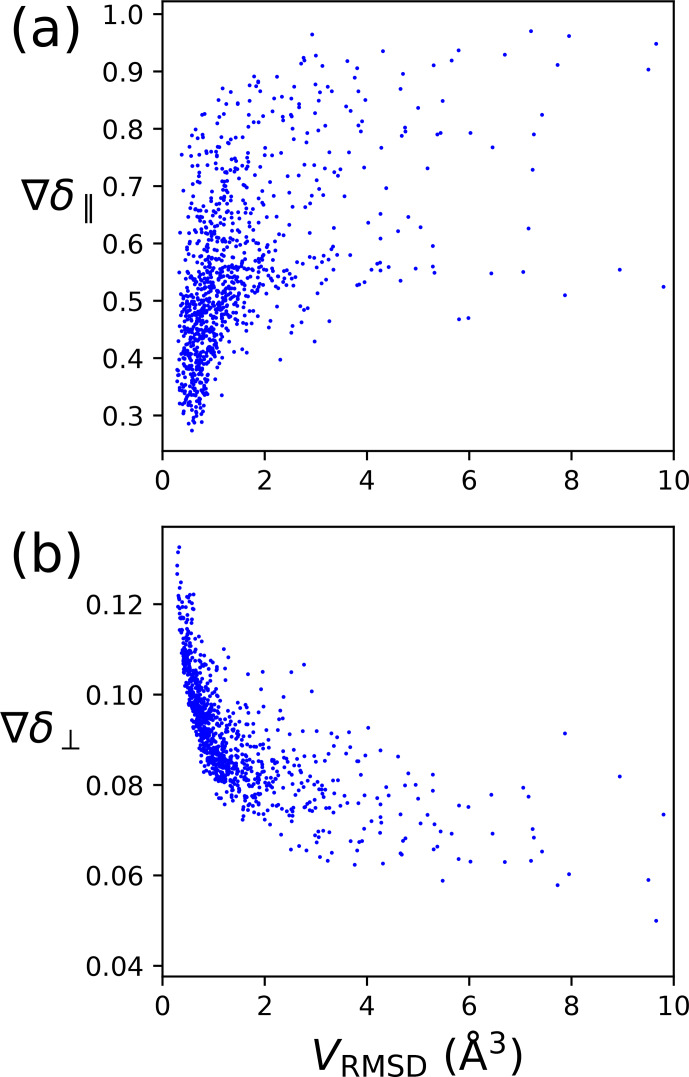
PCS orthogonality metrics versus localisation volumes determined by PCS RMSD. The calculations were performed for the model of four tags at a single site, with the SoI located 20 Å from the paramagnetic centre. Each data point refers to a different set of relative Δχ tensor orientations. **(a)** Parallel metric. **(b)** Perpendicular metric.

## 3 Ubiquitin case study

To verify the performance of four different tags attached at a single site experimentally, we produced samples of the uniformly ${}^{15}$ N-labelled ubiquitin mutant S57C and tagged the cysteine residue with four different thulium tags. The tags were the C1-Tm ${}^{3+}$ [Bibr bib1.bibx10], C2-Tm ${}^{3+}$ [Bibr bib1.bibx8], C12-Tm ${}^{3+}$ [Bibr bib1.bibx13], and C13-Tm ${}^{3+}$ tags, where the C2 and C13 tags are the opposite enantiomers of the C1 and C12 tags, respectively (Fig. [Fig Ch1.F5]). The C13 tag was synthesised for this work, following the general protocol published previously for the C12 tag. In addition, diamagnetic references were generated by tagging the protein with the respective diamagnetic tags loaded with Y ${}^{3+}$ ions. The programme Paramagpy [Bibr bib1.bibx34] was used to determine the Δχ tensors from the PCSs measured for backbone amide protons in [ ${}^{15}$ N, ${}^{1}$ H]-HSQC spectra by fitting to the crystal structure 1UBQ [Bibr bib1.bibx51]. The PCSs and Δχ tensors are reported in Tables S1 and S2, respectively, in the Supplement. The quality factors of the Δχ tensor fits were very good, ranging between 0.014 and 0.019. The paramagnetic centres of all four tags were positioned between 7.6 and 8.3 Å from the C ${}^{\beta }$ atom of the residue in position 57, which is in good agreement with expectations based on the covalent structures of the tags.

**Figure 5 Ch1.F5:**
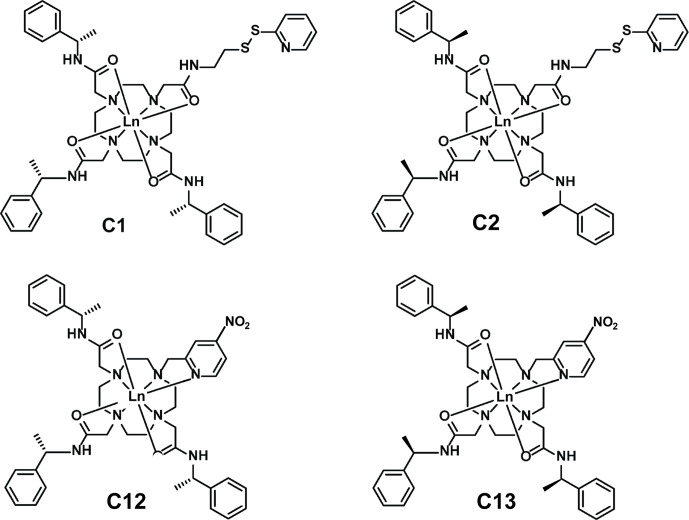
Chemical structures of the lanthanoid tags used in the present work. Compared to the C1 and C12 tags, the C2 and C13 tags have the opposite chirality in each of the phenylethylamide pendants. The C1 and C2 tags react with cysteine thiol groups with the formation of a disulfide bond [Bibr bib1.bibx10], whereas the C12 and C13 tags produce a thioether bond by nucleophilic substitution of the nitro group [Bibr bib1.bibx13].

Figure [Fig Ch1.F6] illustrates the Δχ tensors obtained, depicting the associated PCS isosurfaces for PCSs of ± 1 ppm. The tensors associated with the C12 and C13 tags are larger than those obtained with the C1 and C2 tags, which can be attributed to the conformationally more rigid tether between protein and metal complex (Fig. [Fig Ch1.F5]). The z axes of the Δχ tensors (indicated by the blue isosurfaces) are oriented differently, but, except for the C12 tag, their relative orientations bear a degree of similarity. In particular, the angle between the z axes of the tensors associated with the C2 and C13 tags happened to be quite small (7 ${}^{\circ }$ ), but, with tensor origins differing by 4.8 Å and different rhombicities, the respective PCS isosurfaces would intersect anyway. Similarly, despite their attachment to the same position in the amino acid sequence of ubiquitin, the Δχ tensor fits positioned the paramagnetic centres of different tags somewhat differently, with a distance of 3.7 Å between the lanthanoid ions in the C1 and C2 tags and 7.2 Å in the C12 and C13 tags.

**Figure 6 Ch1.F6:**
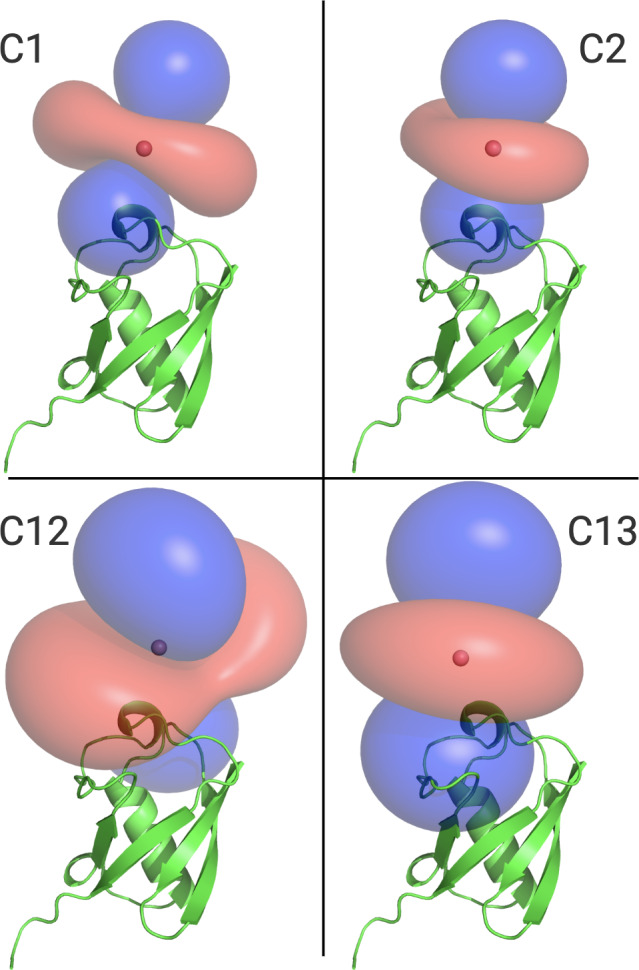
Ubiquitin protein data bank (PDB) model 1UBQ, with PCS isosurface plots for C1, C2, C12, and C13 tags, as shown. Surfaces shown in blue and red represent a constant PCS value of + 1.0 and - 1.0 ppm, respectively. Each panel displays the protein structure in the same orientation.

To assess the capacity of the PCSs to define the correct localisation spaces, we determined the localisation spaces of backbone amide protons in three selected polypeptide segments A–C of ubiquitin, comprising residues 12–17, 29–36, and 64–68, respectively. They were selected because most of the amide protons in these segments were characterised by PCS data measured with all four different paramagnetic tags. All three segments featured $\delta_{⟂}$ metrics below 0.06 (Fig. [Fig Ch1.F7]; Table S3), i.e. lower than required for predicting tight localisation spaces with confidence. Nonetheless, the amide protons with perpendicular metrics >0.025 generally were associated with localisation spaces that closely trace the crystal structure (Fig. [Fig Ch1.F8]).

**Figure 7 Ch1.F7:**
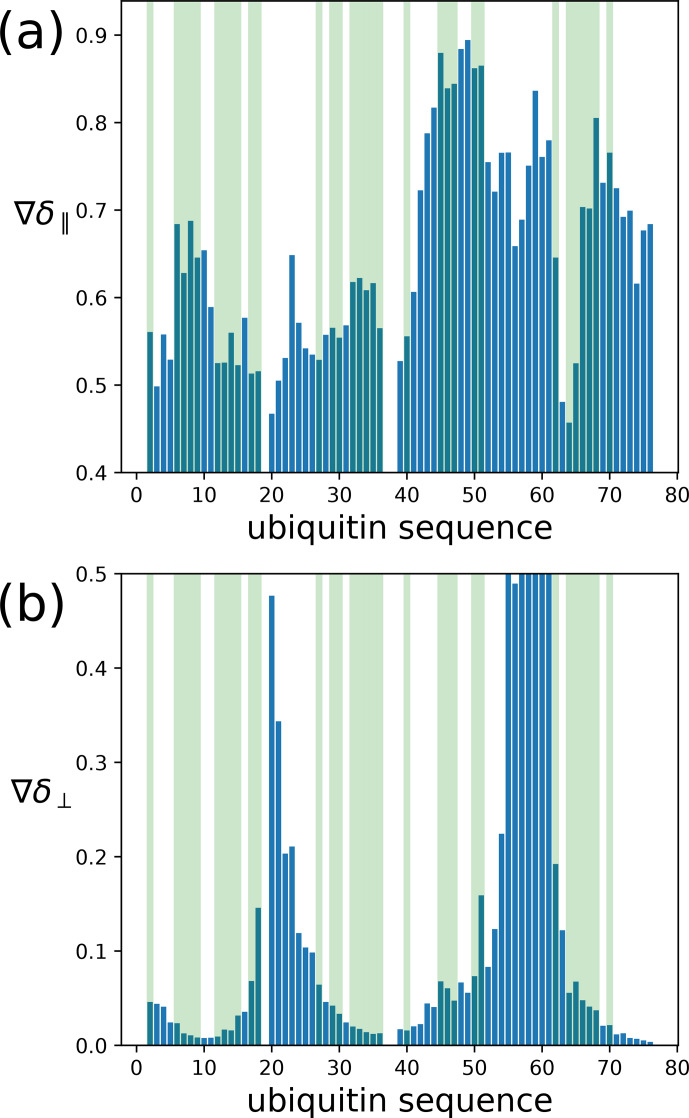
Orthogonality metrics at the sites of different backbone amide protons in the ubiquitin mutant S57C, as defined by the PCSs measured in samples made with four different tags attached to the cysteine residue. **(a)** The $\delta_{∥}$ metric versus amino acid sequence. Light green shading identifies the amino acid residues for which a PCS value could be obtained with all four Tm ${}^{3+}$ tags. **(b)** Same as panel **(a)** but for the $\delta_{⟂}$ metric.

**Figure 8 Ch1.F8:**
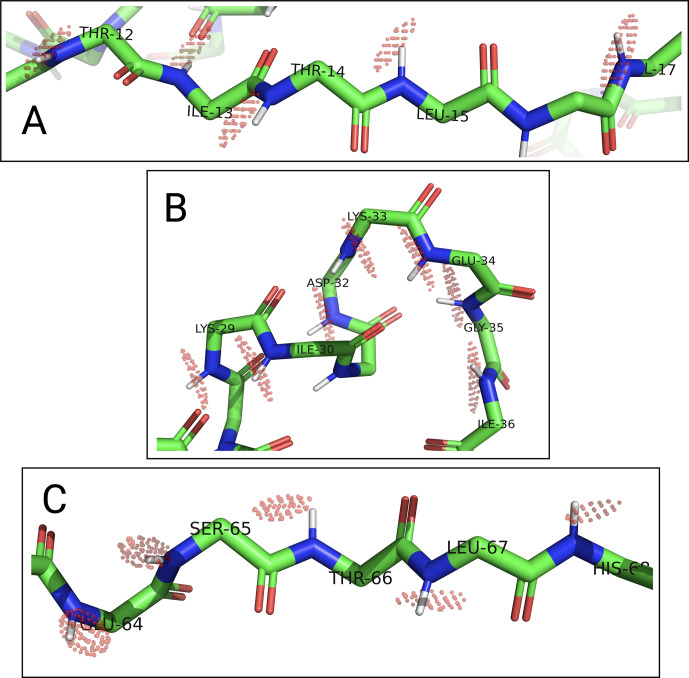
Localisation spaces of backbone amide protons in segments A–C of ubiquitin S57C. The $\delta_{\text{RMSD}}^{\text{thresh.}}$ values of individual amide protons were adjusted to produce similar localisation volumes $V_{\text{RMSD}}$ . Localisation spaces are shown only for amide protons, for which four PCS values were available. Except for Lys33, the centre of each localisation space shown is less than a bond length from the position of the respective amide proton.​​​​​​​

Among the segments A–C, the match between localisation spaces and crystal structure is least satisfying for residues 32–35. To assess the possibility of an artefact arising from PCS isosurfaces intersecting at particularly shallow angles, we calculated the pairwise intersection angles of the isosurfaces for all segments shown in Fig. [Fig Ch1.F8] (Table S4). The smallest intersection angle in segment B was found for Ile36 (11 ${}^{\circ }$ ), but even smaller intersection angles occurred in segments A and C. Furthermore, as the loop region with residues 32–35 at the C-terminus of the α helix is far from the tagging site, it is unlikely that the presence of the tags affected the structure of the loop. Interestingly, however, this loop is known to be flexible and low order parameters have been reported [Bibr bib1.bibx22]. In agreement with this view of conformational disorder, the localisation spaces suggest that the loop is slightly more open than in the crystal structure.

In general, the uncertainties associated with the localisation spaces depend on the reliability of the Δχ tensors, which depend on the accuracy with which PCSs can be measured, the reliability of the protein structure used to fit the Δχ tensors, and the validity of the assumption that each set of PCS data can be fitted reliably by a single effective Δχ tensor. As a first step towards estimating the boundaries of the localisation spaces, a PCS RMSD value can be defined which corresponds to the root mean square deviations (RMSDs) between the experimental PCS values and the PCS values back-calculated at different points in space. The localisation spaces shown in Fig. [Fig Ch1.F8] comprise the points below a certain PCS RMSD threshold which, for illustration purposes, was chosen individually for each amide proton to display localisation spaces of similar volume. These plots therefore trace the general shapes of the localisation spaces and indicate the points of best fit, rather than attempting to give a quantitative account of the respective uncertainties. In each polypeptide segment, the shapes of the localisation spaces are elongated in more or less the same direction, which can be attributed to PCS isosurfaces intersecting at a relatively shallow angle and must not be confused with the shapes of B-factors of crystal structures.

## 4 Discussion

Most of the tags designed for the generation of PCSs in proteins are based on complexes of lanthanoid ions that can be attached to single cysteine residues. Many variants of such single-arm tags have been produced with the aim of generating large PCSs, avoiding multiple tag conformations, producing stable bonds with cysteines, and keeping structural perturbations of the tagged proteins to a minimum [Bibr bib1.bibx33]. As shown in the present work, the availability of a variety of tags turns out to be of great benefit for structure determinations of specific sites of interest, as they allow the generation of Δχ tensors of different relative orientations with a single tagging site. The example of the C1 and C12 tags shows that very different Δχ tensor orientations can be obtained if the same lanthanoid complex is attached to the protein via different chemical tethers. In addition, very different Δχ tensor orientations can be obtained simply by changing the chirality of the pendants in the lanthanoid complex, as illustrated by the C12 and C13 tags (Fig. [Fig Ch1.F6]).

The tethers of all single-arm tags are invariably flexible, leading to variable positions of the lanthanoid ions relative to the target molecule and also variable orientations of the lanthanoid ion complexes. In this situation, the PCSs generated by a tag in the target molecule reflect the average of a multitude of different Δχ tensors and Δχ tensor orientations. Interpreting the average PCSs by a single effective Δχ tensor is an approximation that nonetheless delivers meaningful predictions of PCSs of nuclear spins that are located not too far from the nuclear spins used for fitting the effective Δχ tensor [Bibr bib1.bibx47]. The orientation of an effective Δχ tensor is predominantly governed by the steric and chemical environment of the tag and therefore difficult to predict. It has been observed many times, however, that different paramagnetic metal ions generally produce very similar Δχ tensor orientations, if the chemical structure of the tag and tagging site are the same [Bibr bib1.bibx49]. Similar Δχ tensor orientations lead to shallow intersection angles of the respective PCS isosurfaces. In this situation, which can be identified by a very small $\delta_{⟂}$ metric, relatively large localisation volumes are obtained when PCS isosurfaces are expanded into shells to take uncertainties in PCSs and tensor orientations into account. Shallow intersection angles are commonly observed when the same tag loaded with a different paramagnetic lanthanoid ion (such as Tb ${}^{3+}$ versus Tm ${}^{3+}$ ) is deployed at the same tagging site. Previous work, where we used the PCSs from three different tagging sites to determine the location of a solvent-exposed protein loop, showed that the localisation coordinates determined by PCSs were more accurate when all data encompassed PCSs measured with only a single paramagnetic metal ion [Bibr bib1.bibx35]. Therefore, we conducted the experiments with ubiquitin using Tm ${}^{3+}$ tags only.

The difficulty in predicting Δχ tensor orientations means that two tags may accidentally produce Δχ tensors of a very similar orientation. In the case of ubiquitin S57C, this situation arose for the Δχ tensors of the C1 and C2 tags. On the other hand, the Δχ tensor orientations can be significantly different between two tags that differ only in the chirality of the lanthanoid coordination sphere, as in the case of the C12/C13 pair. Our results also suggest that the perpendicular metric of Eq. ([Disp-formula Ch1.E8]) is a very conservative predictor of the accuracy with which localisation spaces are defined by the PCSs. As demonstrated by the example of ubiquitin, a poor perpendicular metric does not exclude the determination of fairly accurate localisation spaces.

The results of the present work are of great practical value, as they demonstrate that a single cysteine mutant of a target protein can be sufficient to determine accurate localisation spaces from PCSs. Most importantly, the accuracy with which the localisation spaces at a site of interest can be determined by PCSs in a multiple-tag/single-site scenario is statistically comparable to that obtained with the same number of tags deployed at different tagging sites. Not all solvent-exposed residues of a protein are suitable tagging sites after mutation to cysteine because of structural importance (e.g. salt bridges, hydrogen bonds, and secondary structure propensities) or functional impacts (such as allosteric effects and solubility), and the effect of mutations is often hard to predict. The ability to work with a single site for tagging can thus be of critical importance, aside from the convenience of working with a single protein construct rather than multiple mutants. In a fortuitous development, an increasing number of different lanthanoid tags have recently been published that are designed for attachment to cysteine residues and deliver different Δχ tensor orientations [Bibr bib1.bibx30]. The present work shows that different enantiomers of the same tag can produce quite different Δχ tensor orientations. Furthermore, the average positions of the lanthanoid ion can differ between different enantiomeric forms of the tag, which increases the likelihood of Δχ tensors intersecting at a steeper angle.

The statistical analysis of the present work suggests that four different tags have a high chance of defining a small localisation space. In the rare situation where four different tags at a single site fail to deliver a sufficiently well-defined localisation space, chances are that a fifth tag deployed at the same site substantially reduces the localisation space and does this better than if it were attached at an alternative tagging site, which may be available only at a less optimal distance from the SoI.

Attaching four paramagnetic tags still requires four different tagging reactions, and in principle, the production of diamagnetic references requires another set of four tagging reactions with the corresponding tags loaded with diamagnetic metal ions. A further simplification may be possible in rigid proteins, however, if the Δχ tensor fits use only PCSs from regions of the protein that are sufficiently far from the tagging site to display conserved chemical shifts with and without a tag. In this case, the protein without a tag may serve as the diamagnetic reference, removing the need to ligate the protein with diamagnetic tags. It is commonly observed that the chemical shift changes introduced by diamagnetic tags are negligible for most of the protein, even when the protein is small [Bibr bib1.bibx27]. An optimal tagging site is well separated from the SoI to minimise any structural perturbation and paramagnetic relaxation enhancements, while generating sizeable PCSs at the SoI that are easily measured and assigned.

The uncertainties of the localisation spaces depend on a number of different factors, some of which are difficult to capture in a rigorous manner. As mentioned above, most tags are flexible, and explaining the PCSs by a single effective Δχ tensor introduces errors for tags undergoing large translational movements of the metal ion relative to the protein. The magnitude of these errors can be controlled, if the PCSs of nuclei near the SoI are well explained by the fit of the effective Δχ tensor [Bibr bib1.bibx47].

Another source of uncertainty is associated with the uncertainty of the protein structure in solution. The structural flexibility of ubiquitin has been documented in great detail [Bibr bib1.bibx9]. Previous work using PCSs and residual dipolar couplings (RDCs) generated by the C1-Tb ${}^{3+}$ tag at different sites established that the different structures of ubiquitin yielded similar quality factors in the Δχ tensor fits, although, by a small margin, the dynamic structure 2KOX fitted best [Bibr bib1.bibx37]. In the present work, we settled on using the crystal structure 1UBQ [Bibr bib1.bibx51] for simplicity.

Spectral overlap and paramagnetic peak broadening present two additional sources of uncertainties, which are easier to quantify. In the case of the ubiquitin data used in this work, the uncertainty of the PCS data associated with the amide protons of segments A–C was small (estimated to be less than ± 0.01 ppm). A straightforward approach that indirectly captures the effect of most of the sources of uncertainty on the localisation spaces relies on performing the Δχ tensor fits multiple times with random omission of a certain percentage of the PCSs [Bibr bib1.bibx35].

In the present work, we did not attempt to investigate the impact of variable Δχ tensor fits on the localisation spaces for the polypeptide segments of ubiquitin shown in Fig. [Fig Ch1.F8], as the protein was only used as a model system to evaluate the potential of using a single tagging site with four different tags. The C1, C2, C12, and C13 tags were the only tags we tested with this ubiquitin mutant, and this set of tags delivered Δχ tensors with suboptimal $\delta_{⟂}$ metrics and some shallow intersections between PCS isosurfaces. Nonetheless, the localisation spaces agreed remarkably well with the crystal structure, as indicated by their close proximity to the coordinates of the respective amide protons (Fig. [Fig Ch1.F8]). More experience with different protein targets and tags will be required to establish the general validity of these observations.

It is well-known that additional data can be extracted from [ ${}^{15}$ N, ${}^{1}$ H]-HSQC spectra, such as residual dipolar couplings (RDCs) arising from paramagnetic molecular alignment in the magnetic field [Bibr bib1.bibx44] and cross-correlated relaxation effects between dipolar interactions and Curie spin relaxation, which also contain structural information that depends on the orientation of the Δχ tensor [Bibr bib1.bibx40]. Furthermore, the [ ${}^{15}$ N, ${}^{1}$ H]-HSQC spectra detect PCSs of ${}^{15}$ N spins, which can be used to determine localisation spaces, provided the effects from residual anisotropic chemical shifts are taken into account [Bibr bib1.bibx14]. The present work focused on PCS data alone, as chemical shifts can be measured with greater sensitivity than other NMR data.

## 5 Conclusions

While the orientations of the Δχ tensors associated with different tag molecules vary and most often cannot be predicted, the statistical predictions of the current work provide helpful guidance for choosing the optimal numbers of tagging sites and tags. In the case of a single selected site of interest, such as the conformation of a loop region, the binding site of a ligand or a protein–protein interface, our results indicate that PCSs generated with a single tagging site and four different tags most likely yield structural information that is nearly as good as that obtained with a single tag deployed at four different sites. As mutations and chemical modifications can alter the structure and function of a protein and also the NMR resonance assignments at a SoI, it is of great practical advantage if only a single suitable tagging site needs to be identified rather than multiple sites. The benefit applies equally to the use of PCSs for protein resonance assignments based on a known 3D structure of the protein [Bibr bib1.bibx41]. As many different tags have been published in recent years, we believe that the present findings will greatly increase the appeal of strategies based on paramagnetic lanthanoid tags.

## Supplement

10.5194/mr-3-65-2022-supplement The supplement related to this article is available online at: https://doi.org/10.5194/mr-3-65-2022-supplement.

## Data Availability

NMR spectra are available at https://doi.org/10.5281/zenodo.6004596 (Abdelkader, 2022). The script for calculating localisation spaces is available at https://doi.org/10.5281/zenodo.6059659 (Orton et al., 2022b) and from the GitHub site of Paramagpy.
