# Supplementary material for: Localising nuclear spins by pseudocontact shifts from a single tagging site
Source: Magn Reson (Gott). 2022 May 9;3(1):65–76. doi: 10.5194/mr-3-65-2022 (PMC10539793; doi:10.5194/mr-3-65-2022)
Supplement: The supplement related to this article is available online at: https://doi.org/10.5194/mr-3-65-2022-supplement. [file mr-3-65-supplement.pdf]

Supplement of Magn. Reson., 3, 65–76, 2022
https://doi.org/10.5194/mr-3-65-2022-supplement
© Author(s) 2022. CC BY 4.0 License.

Supplement of

Localising nuclear spins by pseudocontact shifts from a single tagging site

Henry W. Orton et al.

Correspondence to: Gottfried Otting (gottfried.otting@anu.edu.au)

The copyright of individual parts of the supplement might differ from the article licence.

Supporting Information

Table of Contents

Synthesis and characterisation of ligand C13 and corresponding Ln complexes

Protocols of expression, purification and tagging of ubiquitin S57C and NMR measurements

Table S1. PCSs of backbone amide protons of ubiquitin S57C with C1, C2, C12 and C13 tags

Table S2. Fitted Δχ-tensor parameters for ubiquitin S57C

Figure S1. Correlations between experimental and back-calculated PCSs

Figure S2. PCS parallel metric versus volume of the localisation space

Figure S3. PCS perpendicular metric versus volume of the localisation space

Table S3. RMSD threshold and characterisation of the localisation spaces of the amide protons shown in Fig. 7 of the main text

Table S4. Angles at which pairs of PCS isosurfaces intersect at the amide protons shown in Fig. 7 of the main text

References

Synthesis and characterisation of ligand C13 and corresponding Ln complexes

2-(Hydroxymethyl)-4-nitropyridine (1)

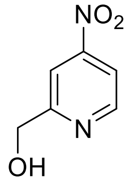

To a solution of commercially available 2-methyl-4-nitropyridine *N*-oxide (0.70 g, 4.5 mmol) in CH_2Cl_2 (14 mL) was added trifluoracetic anhydride (1.2 mL, 9.0 mmol) in CH_2Cl_2 (2.1 mL). The solution was stirred at room temperature for 72 hours, during which time the pale-yellow solution turned deep red in colour. The solvent was removed under reduced pressure and resulting orange oil was dissolved in MeOH (7.0 mL) and saturated aq. K_2CO_3 solution was added until the pH was approximately 8. The solution was stirred at room temperature for 24 hours. The solvent was removed under reduced pressure and the solid was partitioned between water (30 mL) and EtOAc (30 mL) and extracted with EtOAc (2 x 30 mL). The organic layers were combined, dried over magnesium sulfate and concentrated under vacuum to give the crude material, which was purified by column chromatography (silica gel; 0–10 % MeOH in CH_2Cl_2) to give the desired methyl alcohol **1** (0.225 g, 33 %) as a pale-yellow solid. ^{1}H NMR (500 MHz, CD_3OD) δ: 8.77 (d, *J* = 5.4 Hz, 1H), 8.17 (s, 1H), 7.94 (dd, *J* = 5.4 Hz, 1H), 4.78 (s, 2H), O-H signal not observed. ^{13}C NMR (125 MHz, CD_3OD) δ: 165.3, 154.2, 150.9, 114.6, 112.8, 63.9. HRMS (ESI+) found 155.0406 $[M+H]^+$, $[C_6H_7N_2O_3]^+$ requires 155.0451. The NMR data are in agreement with those reported previously (Gempf et al., 2013).

(*R*)-2-Bromo-*N*-(1-phenylethyl)acetamide (2)

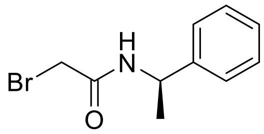

A solution of (*R*)-1-phenylethan-1-amine (6.4 mL, 50 mmol) in anhydrous CH_2Cl_2 (25 mL) was added dropwise to a solution of bromoacetyl bromide (2.2 mL, 28 mmol) in anhydrous CH_2Cl_2 (75 mL), at 0 °C under a nitrogen atmosphere. The reaction mixture was stirred for 2 hours and allowed to warm to room temperature. The reaction mixture was then washed with 2M HCl solution (1 x 60 mL) followed by brine (2 x 60 mL). The organic layer was dried over magnesium sulfate and the solvent was removed under reduced pressure to give pure (*R*)-2-bromo-*N*-(1-phenylethyl)acetamide **2** (0.747 g, 40 %) as a white solid. No further purification

was required. ^{1}H NMR (500 MHz, $CDCl_3$) δ: 7.38–7.27 (m, 5H), 6.77 (s, 1H), 5.14–5.07 (m, 1H), 3.89–3.84 (dd, J = 14.67 Hz, 6.18 Hz, 2H), 1.53 (d, J = 7.00 Hz, 3H). ^{13}C NMR (125 MHz, $CDCl_3$) δ: 164.7, 142.4, 128.9, 127.7, 126.2, 49.7, 29.3, 21.7. HRMS (ESI+): found 263.9995 $[M+Na]^+$, $[C_{10}H_{12}NOBrNa]^+$ requires 264.0000.

2,2′,2″-(1,4,7,10-Tetraazacyclododecane-1,4,7-triyl)tris(*N*-((*R*)-1-phenylethyl)acetamide) (3)

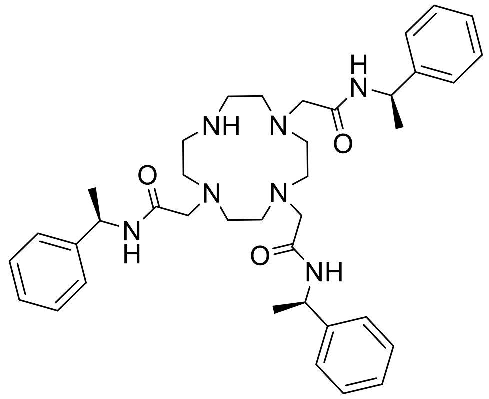

A solution of (*R*)-2-bromo-*N*-(1-phenylethyl)acetamide **2** (0.703 g, 2.90 mmol) in anhydrous CH_3CN (10 mL) was added dropwise to a stirred mixture of cyclen (0.200 g, 1.20 mmol) and potassium carbonate (0.160 g, 1.20 mmol) in anhydrous CH_3CN (50 mL) under a nitrogen atmosphere. The mixture was heated to reflux for 4 hours then allowed to cool to room temperature overnight. Once complete consumption of **2** was observed by LCMS analysis, the reaction mixture was filtered and the solvent was removed under reduced pressure. The residue was taken up in water (300 mL), adjusted to pH 3 and stirred for 30 minutes. EtOAc (150 mL) was added and the biphasic mixture was transferred to a separatory funnel and vigorously shaken. The organic layer containing over-alkylated byproduct was removed. The aqueous layer was then washed with CH_2Cl_2 (2 x 150 mL) and the pH of the aqueous layer adjusted to 5.5 and washed with CH_2Cl_2 (2 x 150 mL). This process was repeated at pH 7. The organic fractions containing desired product were combined, dried over magnesium sulfate and the solvent was removed under reduced pressure to give the desired product (190 mg, 25 %) as a white solid. ^{1}H NMR (400 MHz, CD_3CN) δ: 7.87 (d, J = 7.8 Hz, 1H), 7.74 (d, J = 8.2 Hz, 2H), 7.34–7.17 (m, 15H), 4.98–4.89 (m, 3H), 3.22–3.08 (m, 6H), 2.84–2.47 (m, 16H), 1.40–1.39 (m, 9H). ^{13}C NMR (100 Hz, CD_3CN) δ: 171.6, 171.5, 145.6, 145.4, 129.5, 129.4, 128.0, 127.9, 127.3, 127.0, 60.1, 56.0, 55.1, 53.8, 51.4, 50.0, 49.8, 47.2, 22.8, 22.6. HRMS (ESI+): found 656.4283 $[M+H]^+$, $[C_{38}H_{54}N_7O_3]^+$ requires 656.4288.

Ligand C13

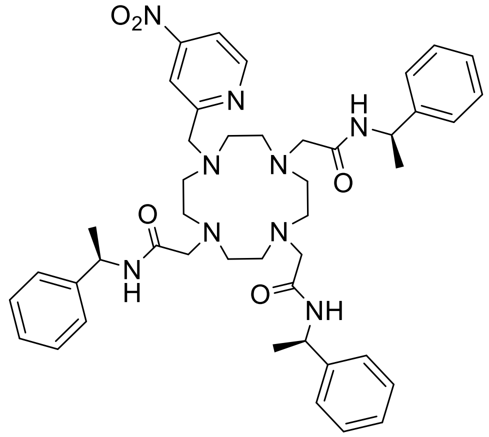

Step 1. To a solution of methyl alcohol **1** (60 mg, 0.38 mmol) and DIPEA (99 µL, 0.57 mmol) in anhydrous CH_2Cl_2 (4 mL) was added methansulfonyl chloride (28 µL, 0.36 mmol) and the resulting mixture was stirred at room temperature for 2 hours. The reaction mixture was washed with brine (10 mL) and the aqueous layer was extracted with CH_2Cl_2 (3 x 10 mL). The combined organic layers were dried over magnesium sulfate and concentrated under reduced pressure to give the mesylate ester (83 mg, 94 %) as a yellow oil, which was used immediately in the next step.

Step 2. To a solution of macrocycle **3** (93 mg, 0.14 mmol) in anhydrous CH_3CN (4.5 mL) was added potassium carbonate (42 mg, 0.30 mmol) and the mixture was stirred for 5 minutes. The mesylate ester of **1** (40 mg, 0.17 mmol) in anhydrous CH_3CN (1.0 mL) was added and the mixture was stirred at 60 °C for 24 hours. The reaction mixture was then cooled to room temperature and centrifuged for 3 minutes. The solution was decanted, and the solid pellet were washed twice with CH_3CN (10 mL). The combined organic layers were concentrated under reduced pressure and the crude material was purified by column chromatography (silica gel; neat CH_2Cl_2 to 9:1 CH_2Cl_2/MeOH) to give the desired ligand **C13** (40 mg, 35 %) as a yellow solid. ^1H NMR (400 MHz, $CDCl_3$) δ: 9.00–7.68 (m, 3H) 8.13 (s, 1H), 7.45–7.05 (m, 17H), 4.87 (t, J = 12.4, 6.2 Hz, 1H), 4.72–4.59 (m, 2H), 3.44–1.97 (m, 2H), 1.44 (t, J = 14.4, 7.0 Hz, 6H), 1.30 (d, J = 7.0 Hz, 3H).^{13}C NMR (100 MHz, $CDCl_3$) δ: 171.4, 170.8, 169.8, 161.3, 153.8, 151.4, 145.3, 144.5, 143.9, 128.5, 128.4, 128.1, 127.3, 126.9, 126.8, 126.6, 126.3, 125.7, 115.7, 114.6, 58.3, 58.0, 56.8, 51.1, 50.8, 50.1, 49.3, 22.8, 22.6, 21.8. HRMS (ESI+): found 814.4373 $[M+Na]^+$, $[C_{44}H_{57}N_9O_5Na]^+$ requires 814.4375.

Ln.C13

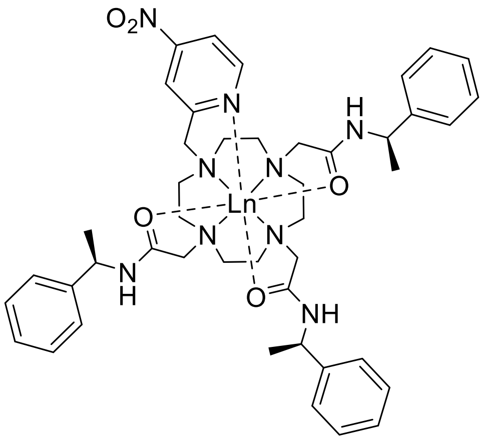

To a solution of **C13** (100 mg, 0.13 mmol) in a mixture of MeCN/H_2O (1:1, 6 mL) was added $LnCl_3.6H_2O$ (1.05 equiv., where Ln = Tm, Y) and the reaction mixture was stirred at 70 °C for 2 hours. Complete complexation was observed by LCMS analysis after this time. The organic solvent was removed under reduced pressure and the water was removed by freeze drying. The crude complexes, **Tm.C13** and **Y.C13**, were obtained as white solids (120 mg, quant.) in each case. The complexes were purified by reverse-phase HPLC [XBridge C18 column, gradient: 0 – 50 % methanol (0.05 % v/v formic acid) in water (0.05 % v/v formic acid), over 10 minutes at 17 mL per minute] to give **Tm.C13** (20.5 mg, 16 %) and **Y.C13** (20 mg, 17 %) as white solids. Analytical RP-HPLC analysis [XBridge C18 column, 100% water (0.1 % v/v formic acid) for 5 minutes followed by a gradient of 0 – 50 % methanol (0.1 % v/v formic acid) over 10 minutes, at 0.7 mL per minute] revealed single peaks at RT = 15.6 minutes for **Tm.C13**, and RT = 11.8 minutes for **Y.C13**. HRMS (ESI+): for **Tm.C13** found 320.1267; $[C_{44}H_{57}N_9O_4Tm]^{3+}$ requires 320.1269; for **Y.C13** found 293.4507; $[C_{44}H_{57}N_9O_4Y]^{3+}$ requires 293.4508.

Protein expression and purification of ubiquitin S57C

Uniformly ^{15}N-labelled human ubiquitin S57C was produced in fusion with a C-terminal $SerHis_6$ peptide. The protein was expressed from a pETMCSI vector (Neylon et al., 2000) in *E. coli* BL21 (DE3) in a bioreactor, using a published high cell-density protocol with cells grown in the minimal fermenter medium containing $^{15}NH_4Cl$ (Klopp et al., 2018). Protein expression was induced with 1 mM isopropyl β-D-1-thiogalactopyranoside and the cells were harvested by centrifugation after expression at 18 °C for 16 h. Following resuspension in buffer A (50 mM Tris-HCl pH 7.5, 300 mM NaCl, 5 % glycerol, 10 mM imidazole), the cells were

lysed using an Avestin Emulsiflex C5 (Avestin, Canada) using two passes at 10,000–15,000 psi. The cell lysate was clarified by centrifugation for 1 h at 30,000 *g*. The supernatant was loaded onto a 5 mL HisTrap FF column connected to an ÄKTA pure 25 chromatography system (Cytiva, USA). The column was washed with 20 column volumes buffer B (same as buffer A but with 20 mM imidazole) and the protein was eluted with 3 column volumes buffer C (same as buffer A but with 500 mM imidazole). The eluted protein was desalted using a HiPrep Desalting 26/10 column (Cytiva, USA) equilibrated with buffer D (50 mM Tris-HCl buffer pH 8.0, 150 mM sodium chloride, 1 mM dithiothreitol (DTT)) followed by removal of the C-terminal $SerHis_6$ peptide with deubiquitinase (Catanzariti et al., 2004). The final yield of purified ubiquitin S57C was 60 mg/L cell culture.

Protein tagging

To a 20 μM solution of ^{15}N-labelled ubiquitin S57C in phosphate-buffered saline (PBS) buffer (137 mM NaCl, 2.7 mM KCl, 10 mM Na_2HPO_4, 1.8 mM KH_2PO_4, pH 7.5) 5 mM DTT was added and the sample was incubated at 25 °C for 1 h. DTT was removed by passing through a PD-10 desalting column (Cytiva, USA) equilibrated with PBS buffer. Immediately following the desalting step, 5 equivalents of the required tag were added and the sample was incubated with shaking at 25 °C for 16 h. Excess unreacted tag was removed by passing through a PD-10 desalting column equilibrated with the NMR buffer (20 mM phosphate buffer, pH 6.5). Finally, the NMR samples were concentrated using 3 kDa molecular weight cut-off Amicon ultrafiltration centrifugal tubes (Merck Millipore, USA) to a protein concentration of 0.2 mM.

NMR measurements

All NMR data were acquired at 35 °C on a Bruker 800 MHz NMR spectrometer equipped with a TCI cryoprobe. The PCSs of the amide protons were measured in [^{15}N,^{1}H]-HSQC spectra recorded with acquisition times of t_{1max} = 39 ms and t_{2max} = 95 ms. PCSs were measured as the chemical shift measured in the sample with Tm^{3+} tag minus the corresponding chemical shift in the sample with Y^{3+} tag.

Table S1. Experimentally measured amide proton PCSs for ubiquitin S57C with C1, C2, C12 and C13 tags.[a]

Residue number	C1 PCS (ppm)	C2 PCS (ppm)	C12 PCS (ppm)	C13 PCS (ppm)
2	-0.099	0.159	0.125	0.108
4	0.111	0.225		0.456
5	0.119	0.172		
6	0.148	0.171	-0.103	0.477
7	0.093	0.107	-0.046	0.311
8	0.101	0.097	-0.052	0.322
9	0.080	0.077	-0.037	0.247
11	0.067	0.077	-0.027	
12	0.055	0.078	-0.012	0.203
13	0.094	0.128	0.001	0.315
14	0.062	0.112	0.070	0.242
15	0.088	0.186	0.128	0.346
17	0.072	0.278	0.550	0.333
18	0.272	0.437	1.456	0.457
20		0.801	4.730	0.163
21	0.807	0.719	2.926	
22				1.007
26	0.491			1.036
27	0.401	0.346	0.410	0.935
29	0.227	0.221	0.428	0.508
30	0.202	0.206	0.274	0.501
31	0.162		0.223	0.442
32	0.121	0.126	0.240	0.313
33	0.102	0.119	0.184	0.271
34	0.096	0.109	0.132	0.275
35	0.088	0.092	0.127	0.253
36	0.106	0.102	0.102	0.294
39	0.146	0.112		0.411
40	0.142	0.113	0.063	0.420
41	0.180	0.142		0.531
42	0.201	0.166		0.641
43	0.345	0.283		1.145
44		0.270	-0.282	
45	0.405	0.355	-0.639	1.426
46	0.265	0.243	-0.547	0.802
47	0.225	0.170	-0.494	0.681
48				1.259
50	0.479	0.396	-0.530	1.817

51	0.839	0.691	-0.878	3.405
52	0.526	0.360		1.579
59		3.224		
61	1.310	2.458		
62	0.092	0.544	-0.821	0.503
64	-0.067	0.179	-0.094	0.185
65	0.013	0.241	-0.243	0.318
66	0.118	0.214	-0.318	0.462
67	0.198	0.254	-0.178	0.663
68	0.251	0.254	-0.273	0.826
69	0.165	0.166	-0.112	
70	0.187	0.164	-0.118	0.600

[a] The PCSs were measured in [^{1}H,^{15}N]-HSQC spectra of uniformly ^{15}N-labelled ubiquitin S57C labelled with one of the four different tags (loaded with Tm^{3+} or Y^{3+} in the paramagnetic and diamagnetic tags, respectively).

Table S2. Fitted $\Delta\chi$-tensor parameters for ubiquitin S57C with C1, C2, C12 and C13 tags.[a]

Tag	$\Delta\chi_{ax}$[a]	$\Delta\chi_{rh}$[a]	*x* (Å)	*y* (Å)	*z* (Å)	α (°)	β (°)	γ (°)	*Q*[b]	*d* (Å)[c]
C1	8.2	2.2	18.729	11.036	11.086	48	61	124	0.019	7.6
C2	7.0	3.0	15.662	13.465	11.630	38	73	1	0.020	8.3
C12	20.1	10.3	20.326	10.342	9.039	9	93	66	0.024	7.8
C13	18.0	4.0	17.797	13.067	15.921	47	76	13	0.017	8.3

[a] $\Delta\chi$ tensor values are given in units of 10^{-32} m^3. Coordinates and Euler angles are reported with respect to the PDB coordinates 1UBQ (Vijay-Kumar et al., 1987). Euler angles are given in the "ZYZ" convention. Tags were loaded with Tm^{3+} or Y^{3+} ions to obtain paramagnetic samples and diamagnetic references, respectively.

[b] *Q* factor calculated as root-mean-square deviation between measured and predicted PCSs divided by the root-mean-square of the measured PCSs.

[c] Distance of the paramagnetic centre from the C^{β} atom of residue 57 in the crystal structure 1UBQ.

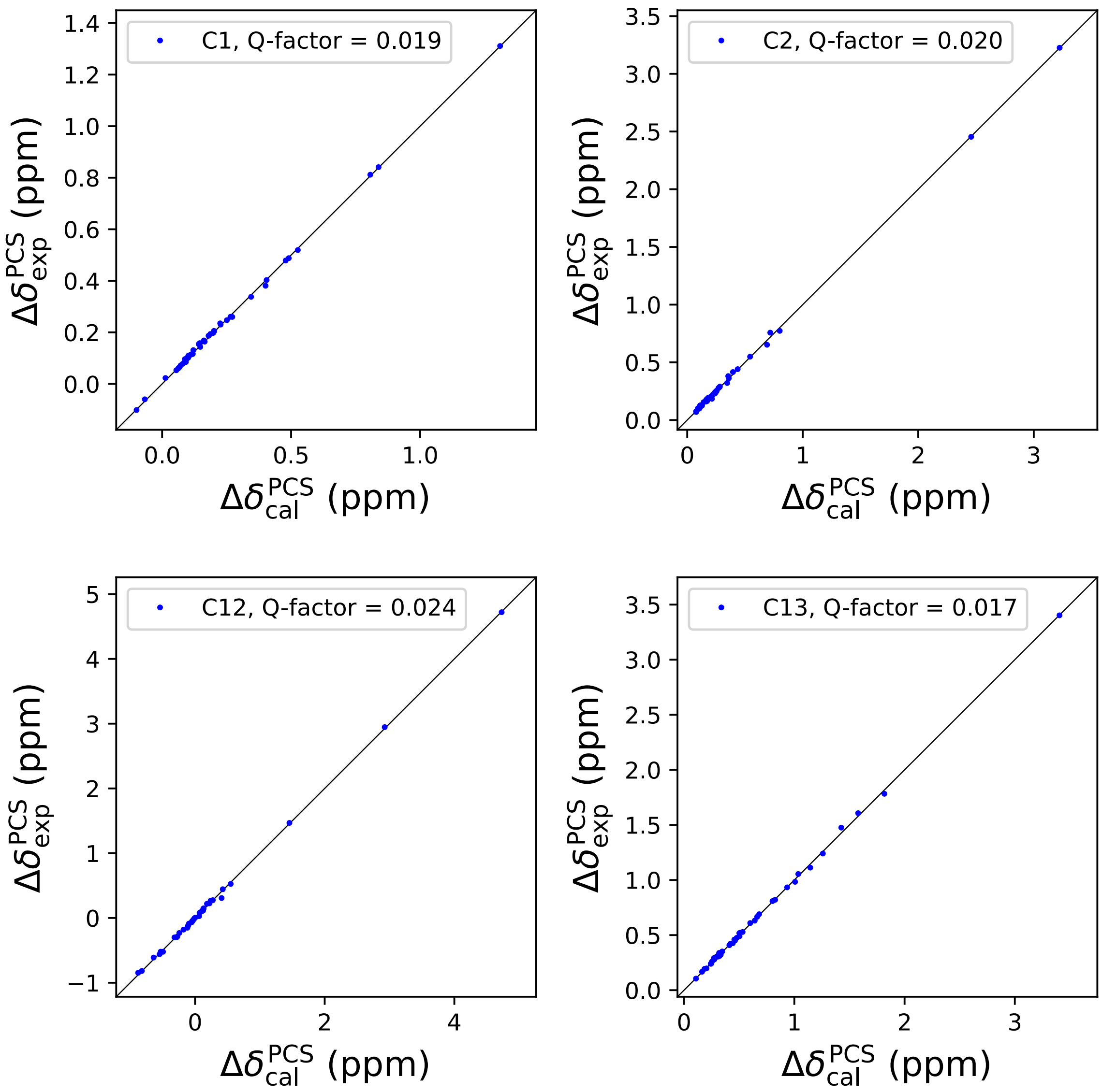

Figure S1. Correlation between experimental and back-calculated PCSs of backbone amide protons in ubiquitin S57C for the $\Delta\chi$-tensor fits of Table S1.

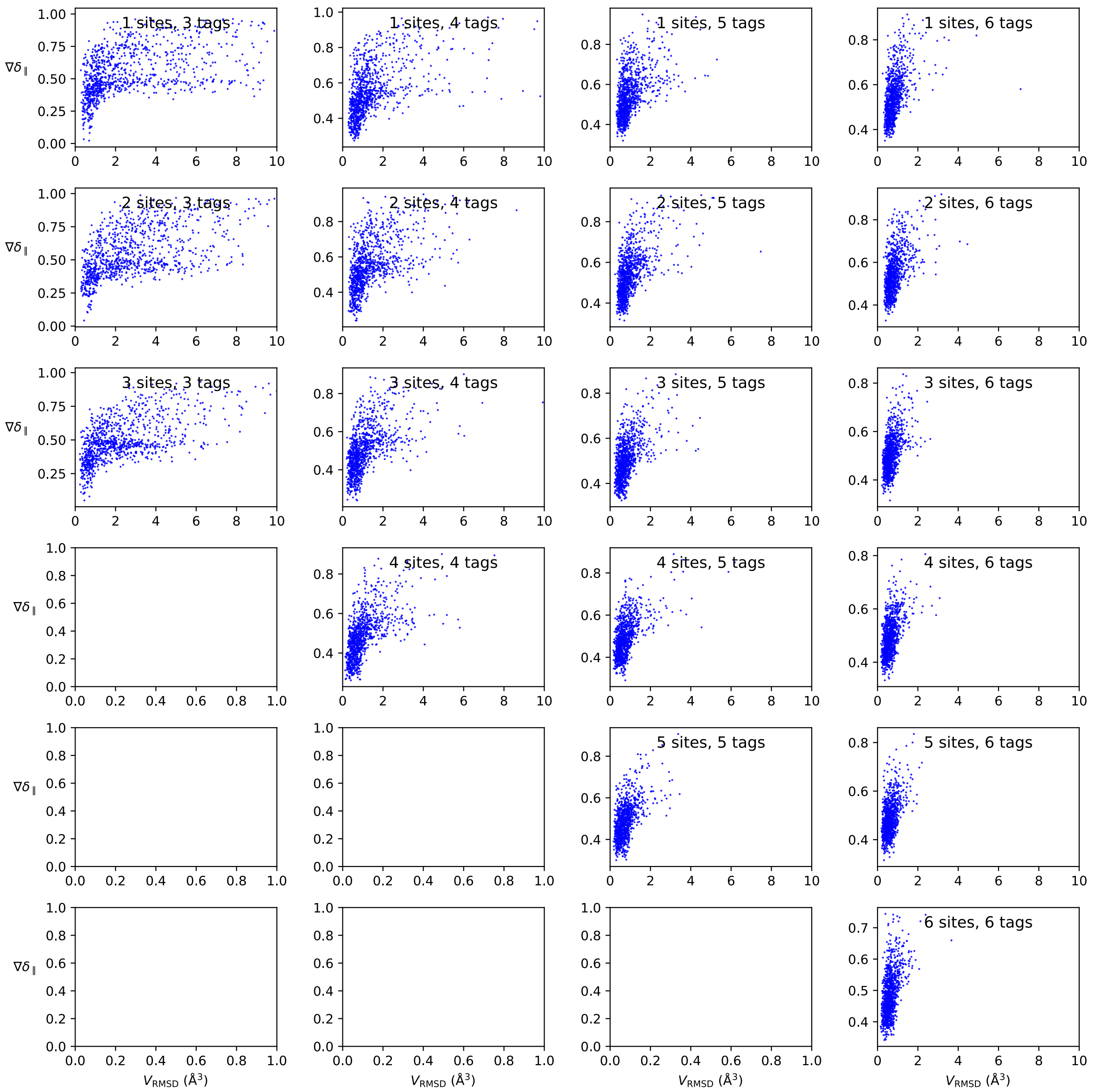

Figure S2. PCS parallel metric versus volume of the localisation space for different numbers of tagging sites and tags. The tagging sites and tag distributions were modelled as described in the main text, with the SoI located at a distance *d* of 20 Å from the paramagnetic centres (Fig. 1). Each plot shows the result of 1000 sampled Δχ-tensor orientations. The correlation between the metric and the localisation spaces improves with the number of tags and tagging sites.

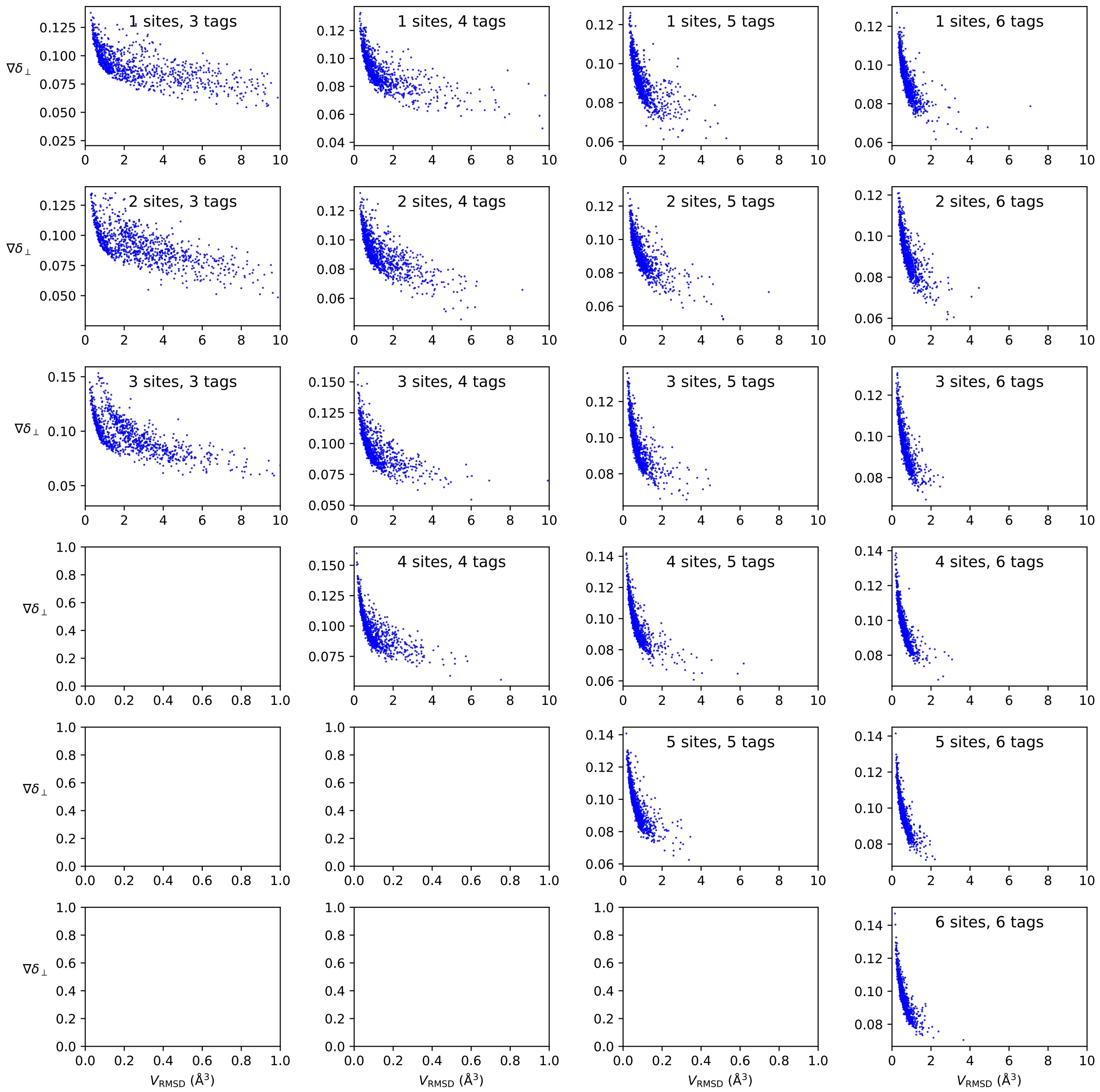

Figure S3. PCS perpendicular metric versus volume of the localisation space. The calculation used the same tag geometries and tag distributions as in Fig. S3.

Table S3. RMSD threshold and characterisation of the localisation spaces of the amide protons shown in Fig. 7 of the main text.

Segment	Residue number	RMSD threshold (ppm)[a]	V_{RMSD} (Å^3)[b]	Electron-nuclear distance (Å)[c]	Distance of nucleus from PCS RMSD minimum (Å)[d]	Dot-product metric	Cross-product metric
A	12	0.003	0.19	32	0.36	0.63	0.008
A	13	0.004	0.18	28	0.54	0.64	0.014
A	14	0.003	0.18	28	0.52	0.68	0.013
A	15	0.007	0.14	23	0.35	0.66	0.026
A	17	0.02	0.47	19	0.78	0.65	0.056
B	29	0.01	0.19	23	0.32	0.70	0.035
B	30	0.008	0.30	25	0.54	0.69	0.028
B	32	0.008	0.22	28	0.75	0.73	0.016
B	33	0.006	0.23	29	1.2	0.73	0.014
B	34	0.004	0.26	30	1.1	0.72	0.011
B	35	0.006	0.26	32	0.62	0.72	0.010
B	36	0.005	0.25	31	1.0	0.68	0.010
C	64	0.023	0.49	19	0.72	0.60	0.046
C	65	0.02	0.29	18	0.28	0.64	0.054
C	66	0.013	0.18	20	0.89	0.74	0.040
C	67	0.013	0.14	21	0.55	0.73	0.034
C	68	0.008	0.10	22	0.43	0.84	0.031

[a] Lowest contour level of the localisation spaces (referred to as δ_{RMSD}^{thresh} in the main text) used to plot the regions shown in Fig. 7 of the main text.

[b] Volume of the localisation space associated with the PCS RMSD threshold given in the preceding column.

[c] Distance of the amide proton from the average $\Delta\chi$-tensor position.

[d] Distance of the amide proton in the crystal structure from the position with the minimal PCS RMSD.

Table S4. Angles at which pairs of PCS isosurfaces intersect at the amide protons shown in Fig. 7 of the main text.[a]

Segment	Residue	C1 *vs* C2	C1 *vs* C12	C1 *vs* C13	C2 *vs* C12	C2 *vs* C13	C12 *vs* C13
A	Thr12	27	96	14	84	15	96
A	Ile13	25	96	11	87	17	99
A	Thr14	34	81	17	69	21	83
A	Leu15	36	82	15	74	26	88
A	Val17	47	76	19	68	38	88
B	Lys29	20	73	19	74	22	91
B	Ile30	20	78	15	76	20	91
B	Asp32	19	70	15	67	19	83
B	Lys33	22	71	14	66	19	81
B	Glu34	20	74	12	68	17	82
B	Gly35	18	73	12	68	16	81
B	Ile36	16	80	11	75	15	87
C	Glu64	54	81	20	84	34	84
C	Ser65	44	99	15	104	29	101
C	Thr66	29	120	11	116	18	118
C	Leu67	23	117	5	113	20	120
C	His68	15	131	2	125	15	132

[a] In degrees. The angles were calculated between the PCS gradient vectors. They differ from the angle score of Zimmermann et al. (2019) by 90°.

References

Catanzariti, A.-M., Soboleva, T. A., Jans, D. A., Board, P. G., and Baker, R. T.: An efficient system for high-level expression and easy purification of authentic recombinant proteins, Prot. Sci., 13, 1331–1339, https://doi.org/10.1110/ps.04618904, 2004.

Gempf, K. L., Butler, S. J., Funk, A. M., and Parker, D.: Direct and selective tagging of cysteine residues in peptides and proteins with 4-nitropyridyl lanthanide complexes, Chem. Commun., 49, 9104–9106, https://doi.org/10.1039/c3cc45875j, 2013.

Klopp, J., Winterhalter, A., Gébleux, R., Scherer-Becker, D., Ostermeier, C., and Gossert, A. D.: Cost-effective large-scale expression of proteins for NMR studies, J. Biomol. NMR, 71, 247–262, https://doi.org/10.1007/s10858-018-0179-0, 2018.

Neylon, C., Brown, S. E., Kralicek, A. V., Miles, C. S., Love, C. A., and Dixon, N. E.: Interaction of the *Escherichia coli* replication terminator protein (Tus) with DNA: A model derived from DNA-binding studies of mutant proteins by surface plasmon resonance, Biochemistry, 39, 11989–11999, https://doi.org/10.1021/bi001174w, 2000.

Orton, H. W., Herath, I. D., Maleckis, A., Jabar, S., Szabo, M., Graham, B., Breen, C., Topping, L., Butler, S. J., and Otting, G.: Localising individual atoms of tryptophan side chains in the metallo-β-lactamase IMP-1 by pseudocontact shifts from paramagnetic lanthanoid tags at multiple sites, Magn. Reson., 3, 1–13, https://doi.org/10.5194/mr-3-1-2022, 2022.

Vijay-Kumar, S., Bugg, C. E., and Cook, W. J.: Structure of ubiquitin refined at 1.8 Å resolution, J. Mol. Biol., 194, 531–544, https://doi.org/10.1016/0022-2836(87)90679-6, 1987.

Zimmermann, K., Joss, D., Müntener, T., Nogueira, E. S., Schäfer, M., Knörr, L., Monnard, F. W., and Häussinger, D.: Localization of ligands within human carbonic anhydrase II using ^{19}F pseudocontact shift analysis, Chem. Sci., 10, 5064–5072, https://doi.org/10.1039/c8sc05683h, 2019.
